# Transcriptome sequencing reveals core regulation modules and gene signatures of Zusanli acupoints in response to different moxibustion warm stimulation in adjuvant arthritis rat

**DOI:** 10.1186/s41065-022-00221-4

**Published:** 2022-02-22

**Authors:** Li Zeng, Jing Guo, Ping Du, Shuguang Yu, Haiyan Yin

**Affiliations:** 1 Department of Rehabilitation, Medical Center Hospital of QiongLai City, Chengdu, 611530 China; 2 Department of Acupuncture and Tuina, Chengdu University of Traditional Chinese Medicine, Chengdu, 610075 China

**Keywords:** Rheumatoid arthritis, Zusanli acupoint, Moxibustion warm, Hsp90aa1

## Abstract

**Background:**

The efficacy of moxibustion in treating rheumatoid arthritis is recognized, but its molecular mechanism is still unclear. This study aimed to characterize the molecular map and potential key genes in the process of different moxibustion warm at Zusanli acupoint treatment of adjuvant arthritis (AA) model.

**Methods:**

AA rat model was induced by complete Freund’s adjuvant (CFA) and then accessed by foot swelling and thermal hyperalgesia test. Transcriptome sequencing, series test of cluster (STC) and weighted gene co-expression network analysis (WGCNA) were used in this study.

**Results:**

CFA-induced inflammation, foot swelling, and pain in AA rats were significantly improved by moxibustion warm. Differentially expressed genes (DEGs) were screened in nine different comparison groups and a total of 4535 DEGs were identified, and these DEGs were preferentially clustered in inflammatory and immune-related pathways, such as MAPK signaling pathway. Only 1 DEG of heat shock protein 90, alpha (cytosolic), class A member 1 (Hsp90aa1) was shared in comparison groups of model with moxibustion treatment. STC analysis also revealed that Hsp90aa1 was increased in AA model, but decreased after 37 °C moxibustion intervention, and constantly decreased after 42 °C moxibustion treatment. GO and KEGG pathway analysis revealed that these genes enriched in inflammatory and immune-related pathways. Moreover, WGCNA identified that violet module was positively correlated with model temperature while negatively correlated with control, and the paleturquoise module was positively correlated with model. The violet and paleturquoise module gene were significantly enriched in MAPK signaling pathway. Importantly, Hsp90aa1 also played a central role in the violet module by interacting with multiple proteins.

**Conclusions:**

Moxibustion warm improved AA in rat, and we obtained the transcriptome profile and excavate a critical gene of Hsp90aa1, and provided insight into gene signatures for moxibustion warm at Zusanli acupoint in AA rat.

**Supplementary Information:**

The online version contains supplementary material available at 10.1186/s41065-022-00221-4.

## Introduction

Rheumatoid arthritis (RA) is a chronic systematic and autoimmune disorder characterized by destructive joint development [1]. RA has a serious negative impact on patients’ daily activities and quality of life and has a high mortality. Although there are a variety of effective drug treatments for RA, patients still need better treatment methods to reduce pain and financial burden [2]. As far as we know, one of the reasons for the limitation of RA treatment may be that the etiology and pathogenesis of RA are still largely unclear.

In Traditional Chinese medicine, RA belongs to the category of joint pain, and moxibustion is regarded as an important practical means for the clinical treatment of RA. Previous studies have shown that moxibustion on Zusanli acupoint (located at 5 mm below the capitulum fibulae and lateral to the anterior tubercle of the tibia) has a potential effect on RA and can effectively improve the symptoms of RA, and has been used in many clinical studies. For instance, moxibustion at Zusanli acupoint has an analgesic effect in RA rat induced by complete Freund’s adjuvant (CFA) [3]. Moxibustion at Zusanli acupoint also has a potent antinociceptive effect and could modulate neuronal excitability in an arthritic rat [4]. However, the molecular expression profile and key genes of Zusanli acupoint in response to moxibustion therapy for RA remain to be further explored.

In this study, adjuvant arthritis (AA) model rat was induced by CFA, and rat received or did not receive different moxibustion warm treatment at Zusanli acupoint were subjected to transcriptome sequencing to screen for differentially expressed genes (DEGs). The DEGs between different moxibustion warm treatment groups were used for co-expression network analysis to mine the core modules and genes related to moxibustion treatment. Our study profiled the transcriptome landscape and expounded core gene co-expression network and the gene characteristics of the Zusanli acupoint response to moxibustion warm stimulation in AA rats.

## Materials and methods

### Experimental rats and groups

The 36 clean and healthy male SD rats aged 3–4 months with an average weight of 200 ± 20 g were provided by the Experimental Animal Center of Chengdu University of Traditional Chinese Medicine. The temperature and humidity of the breeding environment for all rats were maintained at about 20 °C and 70%, respectively, and the day-night cycle of light was controlled with 12/12 h, and rats were free to drink and eat. The rats were randomly grouped into six groups (six rats per group) and modeled after 1 week of adaptive feeding. The groups were as follows: control group (Con), model group (Model), 37 °C control moxibustion group (CM37), 42 °C control moxibustion group (CM42), 37 °C model moxibustion group (MM37), 42 °C model moxibustion group (MM42). All rats experiment were approved by the Ethics Committee of Experimental Animals of the Qionglai Medical Center Hospital.

### AA model construction and therapeutic intervention

For rats in all model groups, modeling was performed after 1 week of adaptive feeding. The rat model of AA was induced by intracutaneously infusing 0.1 mL of CFA into the pad of the right posterior foot. On the 8th day after modeling, the therapeutic intervention could begin. Except for the rats in the Con group and the Model group, the rats in the other groups were all subjected to moxibustion. The moxibustion stimulation with a corresponding temperature of 37 °C or 42 °C at the Zusanli acupoint (ST36, is located approximately 5 mm below the fibular head and lateral to the anterior tubercle of the tibia) was applied with a small animal moxibustion strip for 15 min once a day. The stick of moxibustion needles was 0.25 × 40 mm and the acupuncture point was 5 mm in depth using a constant electrical character (2 Hz, 2 mA). In the process of moxibustion intervention, a digital temperature recorder was used to detect the temperature of the moxibustion site during and before and after moxibustion, and the target temperature was obtained by adjusting the distance between the moxibustion strips and the acupoint. The rats in the Con group and the Model group were not treated with acupuncture, but went through the same procedure such as the same fixation, time, and method. After stopping the intervention, rats in each group were anesthetized intraperitoneally with 3% sodium pentobarbital (30 mg/kg). Local tissue at the Zusanli acupoint of the rat was collected, and tissues were quickly cut into pieces after being rinsed with DEPC water, and then put into the frozen storage tube containing 1 mL RNA-later, which was quickly placed in liquid nitrogen and stored at − 80 °C.

### Foot swelling

The arthritis model was evaluated by foot swelling by measuring the hindlimb paw volume. The test was performed by a digit capacity plethysmograph apparatus (Shandong Academy of Medical Sciences Equipment Factory, China). Foot swelling of rat was detected at day 0 before modeling and on the 7th day after CFA injection.

### ELISA analysis

The total concentration of tumor necrosis factor alpha (TNF-α, YIFEIXUE BIOTECH, Nanjing, China NO.YFXER00038) and interleukin-1β (IL-1β, YIFEIXUE BIOTECH, Nanjing, China NO.YFXEM00028) in serum of mice were detected by ELISA kit, according to the instructions of the manufacturer.

### Thermal withdrawal latency

Thermal hyperalgesia was evaluated by thermal withdrawal latency (TWL) using a Plantar Test Apparatus (Hargreaves method, PL-200, Tai Meng, China). The rat was placed in a behavior box on a glass platform with acclimation for 30 min, then mobile heat radiation center point was aimed at the swollen plantar surface of the right hind paw of rat. The upper limit time of Plantar Test Apparatus was set at the 20s, and the light intensity was 10%. Any of following responses were counted as the main factors of pain thresholds according to Hargreaves method [5]: the velocity of the withdrawal reflex; the presence or absence of licking; and the duration of the hind paw withdrawal from the floor. Each measurement of paw TWL was performed 5 times, and the interval between each measurement was at least 5 min, and the average value was taken as the final pain threshold. Paw TWL measurement time: before CFA injection; on the 8th day after modeling and before intervention; after moxibustion warm intervention.

### RNA isolation and transcriptome sequencing

Total RNA was isolated from local tissue at the Zusanli acupoint using the TRIzol reagent (Thermo Scientific, USA) according to the manufacturer’s protocol. RNA purity and quantification were evaluated using the NanoDrop 2000 spectrophotometer (Thermo Scientific, USA). Then RNA was reverse-transcribed into cDNA and amplified into the libraries using TruSeq Stranded mRNA LT Sample Prep Kit (Illumina, San Diego, CA, USA) according to the manufacturer’s instructions. The transcriptome sequencing and analysis were commissioned to OE Biotech Co., Ltd. (Shanghai, China). Briefly, the libraries were subjected to an Illumina HiSeq 2500 Ten platform with 150 bp paired-end reads.

### Bioinformatics analysis

Raw data (raw reads) of fastq format were firstly processed using Trimmomatic [6] and the low quality reads were removed to obtain the clean reads. Reads per kilobase of exon per million reads mapped (FPKM) of each gene was calculated using Cufflinks [7], and the read counts of each gene were obtained by HTSeq-count [8]. Differential expression analysis was performed using the DESeq (2012) R package. *P* value < 0.05 and foldchange > 2 or foldchange < 0.5 was set as the threshold for significantly differential expression. Hierarchical cluster analysis of DEGs was performed to demonstrate the expression pattern of genes in different groups and samples. Gene Ontology (GO) enrichment and Kyoto Encyclopedia of Genes and Genomes (KEGG) pathway enrichment analysis of DEGs were performed respectively using R based on the hypergeometric distribution.

### WGCNA analysis

The gene co-expression network were performed using WGCNA package in R [9]. The parameters was set as the standard deviation of expression fluctuation was higher 0.5 among each group. The Pearson correlation coefficient was used to calculate the correlation coefficient and *P*-value of module feature genes and characters. Modules that have different traits were elected out with the absolute value of correlation coefficient > 0.5 and *p*-value< 0.05.

### Statistical analysis

Data analysis was performed by SPSS 12.0 software. Data are expressed as the mean ± standard deviation (SD). One-way ANOVA following Tukey’s test was used for inter-group comparison, and t test was used for two group comparison. *P* < 0.05 was considered a statistically significant outcome.

## Results

### Moxibustion warm improved inflammation and thermal hyperalgesia

**Fig. 1:**
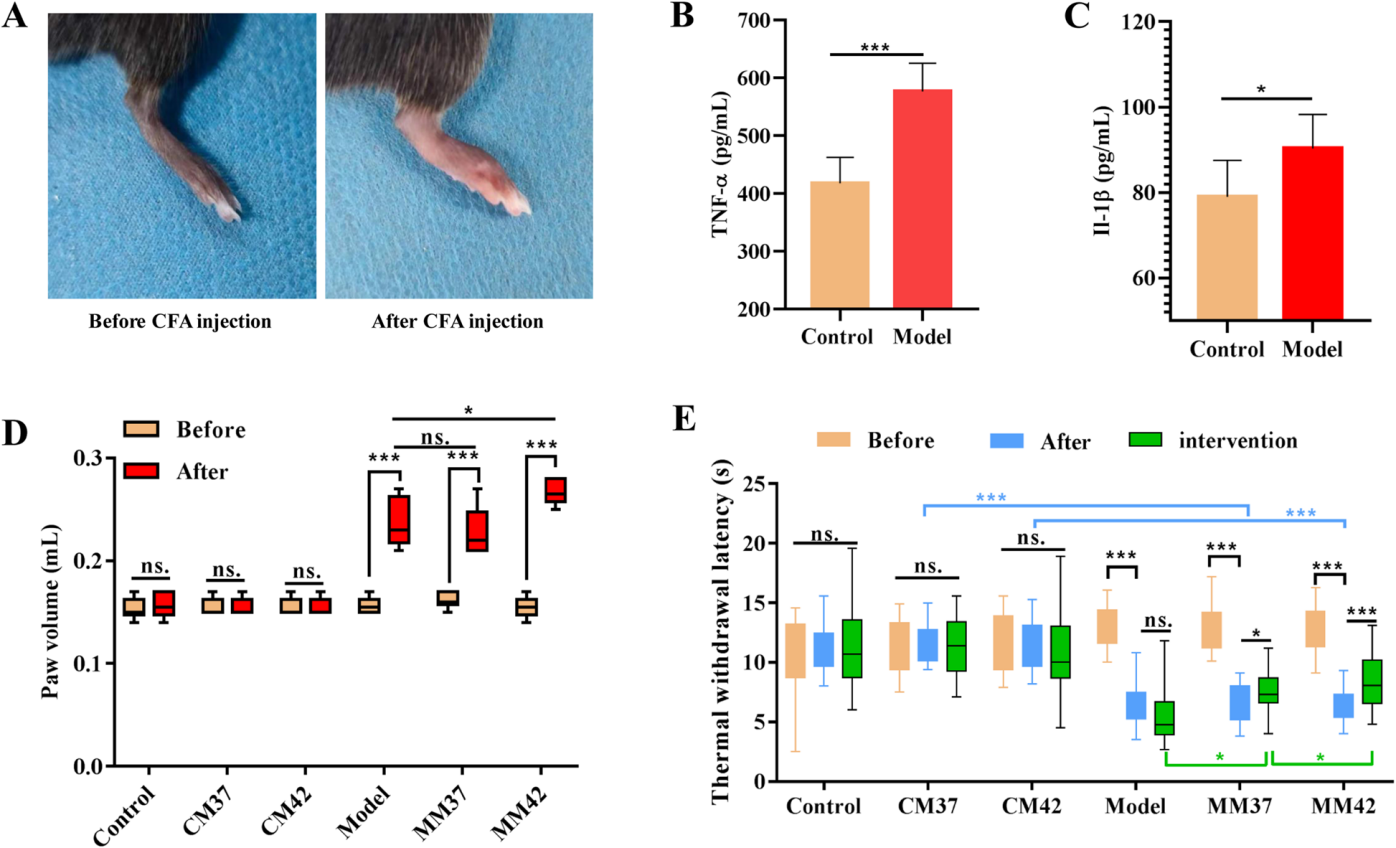
Effect of moxibustion warm on thermal hyperalgesia in rats with AA. **A** A representative image of a swollen rat toe. **B** The content of TNF-α in rat detected by ELISA. **C** The content of IL-1β in rat detected by ELISA. **D** Paw volume of rat with AA detected at before and after CFA induction. **E** Effect of moxibustion on pain threshold of AA rat detected at before and after CFA induction, and after intervened with moxibustion warm. * indicates *P* < 0.05, *** indicates *P* < 0.001, ns. indicates *P* > 0.05

To verify the therapeutic effect of moxibustion on AA, we used CFA treatment to construct AA disease model in rat. As shown in Fig. 1A, on the 8th day after CFA injection, the rat showed visible toe swelling. In addition, the levels of paw inflammatory cytokines of TNF-α (*P* = 0.0002) and IL-1β (*P* = 0.0382) were significantly increased in the model group (Fig. 1B and C). Further, we quantified the degree of toe swelling and measured the thermal hyperalgesia of rat in each group to evaluate whether the AA model was successfully constructed. In the three control group, there was no change in the foot swelling of rats before and after modeling (Fig. 1D). However, in the three model groups, compared with before modeling, the volume of rat foot increased significantly after CFA induction (Model: Before vs after, *P* = 1.71e-05; MM37: Before vs after, *P* = 6.99e-05; MM42: Before vs after, *P* = 1e-08) (Fig. 1D), indicating that the AA model was successful construction and can be used for further experiments. Furthermore, TWL was detected to evaluate the effect of moxibustion warm on thermal hyperalgesia of AA rat. Similarly, in the three control group, there was no difference in TWL at three time points (Fig. 1E). As expected, in the three model group, TWL decreased significantly after CFA induction compared to pre-modeling (Model: Before vs after, *P* = 1.5e-22; MM37: Before vs after, *P* = 5.4e-18; MM42: Before vs after, *P* = 9.4e-19). In MM37 group (*P* = 0.022) and MM42 group (*P* = 4e-05), moxibustion intervention significantly elevated TWL compared to after CFA induction, and the effect of treatment of moxibustion temperatures 42 °C was more significant (Fig. 1E). Moreover, after moxibustion intervention, compared with model group, TWL was significantly elevated in MM37 group (Model vs MM37, *P* = 3.28e-04), and was further significantly increased in MM42 group (MM37 vs MM42, *P* = 0.0012) (Fig. 1E). Therefore, these results indicate that CFA-induced arthritis in rats, moxibustion warm treatment improved the thermal hyperalgesia of AA rats, and the effect of moxibustion temperature 42 °C was more significant than that of moxibustion temperature 37 °C.

### Overall of transcriptome profile of AA rats

Based on the results of toe swelling degree, the samples from groups of Con (*n* = 6), Model (*n* = 5), CM37 (n = 6), CM42 (n = 6), MM37 (n = 6), and MM42 (n = 6) were subjected to transcriptome sequencing. Thus, in our study, 35 samples were sequenced with reference transcriptome, and a total of 258.83G of clean data was obtained. The effective data volume of each sample was distributed from 6.11 to 8.07 G, Q30 bases were distributed between 93.92 and 94.92%, and the average GC content was 49.67% (Supplementary Table 1). Clean reads were compared to the reference genome to obtain the genome alignment of each sample, the results showed that the ratio of mapped reads was ranged from 97.87 to 98.88% (Supplementary Table 1). These results indicated that transcriptome sequencing data was sufficiently reliable.

### Analysis of DEGs of moxibustion at Zusanli acupoint and control in AA rats

**Fig. 2:**
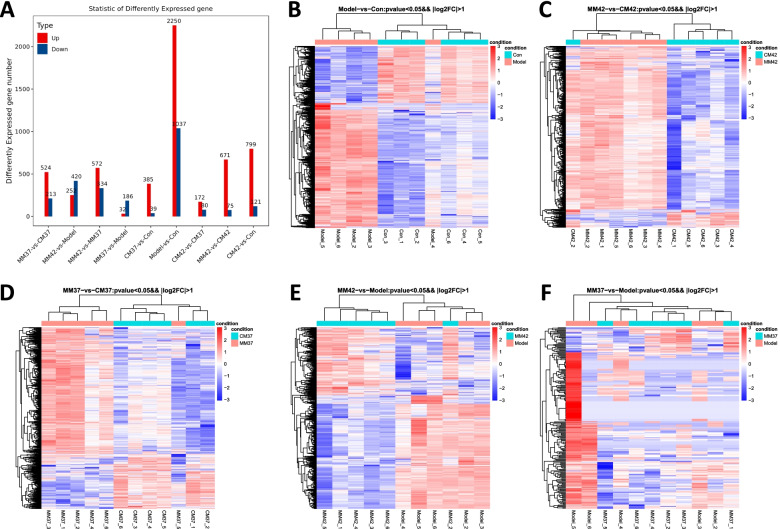
Analysis of DEGs of moxibustion at Zusanli acupoint and control in AA rats. **A** Histogram of the number of DEGs in nine comparison groups. The hierarchical clustering of DEGs between Model and Con (**B**), MM42 and CM42 (**C**), MM37 and CM37 (**D**), MM42 and Model (**E**), MM37 and Model (**F**). The vertical columns represent different groups, the horizontal rows represent DEGs, red represents gene up-regulated and blue represents gene down-regulated

According to the expression levels of protein-coding genes in different samples, DEGs were screened in nine different comparison groups and the total of 4535 DEGs in nine comparison groups was identified. As shown in Fig. 2A, there were 524 up-regulated genes and 213 down-regulated genes in MM37 group compared to CM37 group; there were 671 up-regulated genes and 75 down-regulated genes in MM42 group compared to CM42 group; there are 2250 genes up-regulated and 1037 genes down-regulated in Model group compared to Con group. All these DEGs could be separated into two clusters of AA model or control in hierarchical clustering (Fig. 2B-D). Therefore, CFA induction tends to activate gene expression in the AA rat. Moreover, compared with Model group, 252 up-regulated genes and 420 down-regulated genes in MM42 group, and 32 up-regulated genes and 186 down-regulated genes in MM37 group (Fig. 2A). Similarly, these DEGs were clustered into two clusters of AA model or moxibustion interfering model in hierarchical clustering (Fig. 2E-F). These indicated that moxibustion at Zusanli acupoint tends to suppress gene expression in the AA rat.

**Fig. 3:**
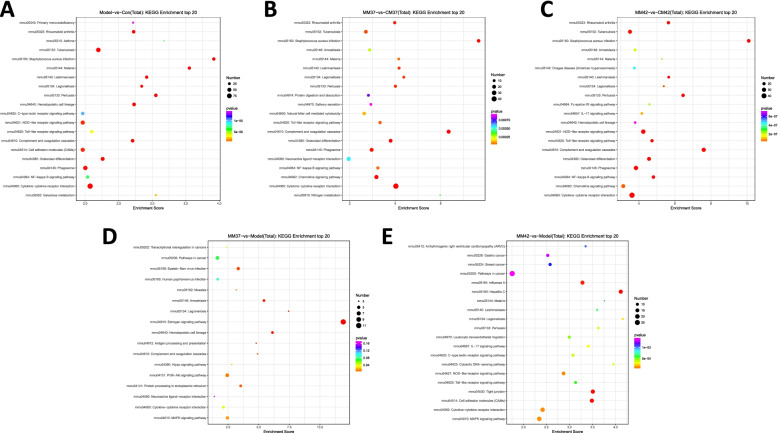
KEGG pathway enrichment of DEGs. KEGG pathway analysis of DEGs between Model and Con (**A**), MM42 and CM42 (**B**), MM37 and CM37 (**C**), MM37 and Model (**D**), MM42 and Model (**E**). The circle size represents the number of DEGs, and the color from red to green indicates a gradual decrease in significance

KEGG pathway analysis showed that DEGs from groups of Model vs Con, MM37 vs CM37, and MM42 vs CM42 were priority enriched into Rheumatoid arthritis pathway (mmu05323) and Tuberculosis (mmu05152), also, DEGs were involved in inflammatory and immune-related pathways, such as Natural killer cell mediated cytotoxicity, IL-17 signaling pathway, Toll-like receptor signaling pathway, NF-kappa B signaling pathway, and Cytokine-cytokine receptor interaction (Fig. 3A-C). Besides, DEGs genes from group of MM37 vs Model and MM42 vs Model also showed similar KEGG enrichment and were also preferentially clustered in inflammatory and immune-related pathways, such as Leukocyte transendothelial migration, IL-17 signaling pathway, Toll-like receptor signaling pathway, Cytokine-cytokine receptor interaction, and MAPK signaling pathway (Fig. 3D-E). These results suggested that CFA induced inflammation in AA rats and subsequent stimulation of moxibustion at Zusanli acupoint activated immune regulation in AA rats.

### Analysis of common and unique DEGs induced by different moxibustion warm at Zusanli acupoint in AA rats

**Fig. 4:**
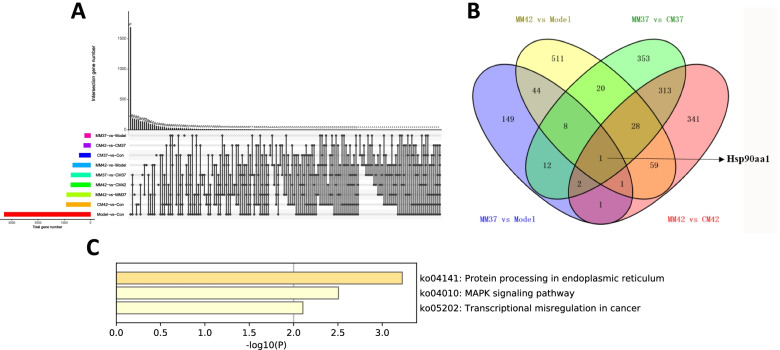
Analysis of common and unique DEGs induced by different moxibustion warm in AA rats. **A** The common and specific DEGs among nine comparison groups. The black dots falling on the same line indicate that there are common DEGs between the comparison groups. **B** Venn diagram of DEGs in different groups. There were only one gene DEG of Hsp90aa1 was shared in four comparison groups. **C** KEGG pathway analysis of 54 common DEGs in four comparison groups. The circle size represents the number of DEGs, and the color from red to green indicates a gradual decrease in significance

In addition, we also counted the number of overlapping DEGs between nine comparison groups (Fig. 4A). As we can see from Fig. 4B, only 1 DEG of heat shock protein 90, alpha (cytosolic), class A member 1 (Hsp90aa1) was shared in groups of MM37 vs CM37, MM42 vs CM42, MM42 vs Model, and MM37 vs Model. Moreover, to explore gene expression dysregulation involved in different moxibustion warm interventions, DEGs in the model group and the different moxibustion warm treatment model group were overlapped (take the intersection of two groups of DEGs). As mentioned above, MM42 vs Model comparison produced 672 DEGs, and MM37 vs Model produced 218 DEGs. However, the overlapping results showed that there were only 54 common DEGs in MM42 vs Model and MM37 vs Model (Fig. 4B), and these 54 DEGs were mainly involved in protein processing in endoplasmic reticulum, MAPK signaling pathway, and transcriptional misregulation in cancer (Fig. 4C). These results indicated that these genes responding to moxibustion therapy may improve AA through the above three pathways.

### Series test of cluster (STC) for moxibustion intervention in AA rat

**Fig. 5:**
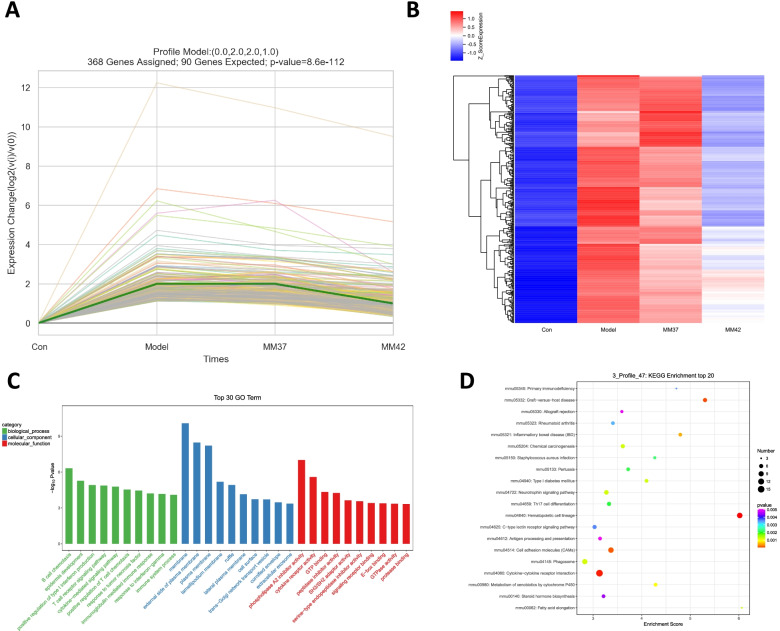
STC analysis for moxibustion intervention in AA rat. **A** The Profile ID 47 contained 368 genes and they increased in Model group and decreased in MM37 and MM42 groups. **B** Trend analysis of DEGs in Profile ID 47. Red represents gene up-regulated and blue represents gene down-regulated. **C** GO functional analysis of DEGs in Profile ID 47. **D** KEGG pathway enrichment of DEGs in Profile ID 47. The circle size represents the number of DEGs, and the color from red to green indicates a gradual decrease in significance

Subsequently, to narrow the gene groups and key genes that play a leading role in the treatment of AA by moxibustion, all DEGs were subjected to STC analysis. Ultimately, we declared 3641 DEGs using STC by 50 model profiles, while only 11 patterns of genes with significant *P* values were identified. According to ascending P value, the most significant pattern was Profile ID 47 (P value = 8.6e-112), which contained 368 genes assigned and 90 genes expected (Fig. 5A). It is worth mentioning that Hsp90aa1 was also observed here. The expression of these genes increased after CFA-induced AA model, but decreased after 37 °C moxibustion intervention, and constantly decreased after 42 °C moxibustion treatment (Fig. 5A and B). The expression pattern in the Profile ID 47 model was consistent with the results in Fig. 1. Moreover, GO functional showed that these genes involved in top ten biological processes were related to immune regulation, including B cells, T cells, and interleukin-mediated regulation (Fig. 5C). KEGG pathway analysis revealed that these genes enriched in inflammatory and immune-related pathways, such as Primary immunodeficiency, Rheumatoid arthritis, and Cytokine-cytokine receptor interaction (Fig. 5D). Therefore, these results indicated that moxibustion improves AA by activating the immune response.

### Core gene co-expression network modules correlated with moxibustion at Zusanli acupoint

**Fig. 6:**
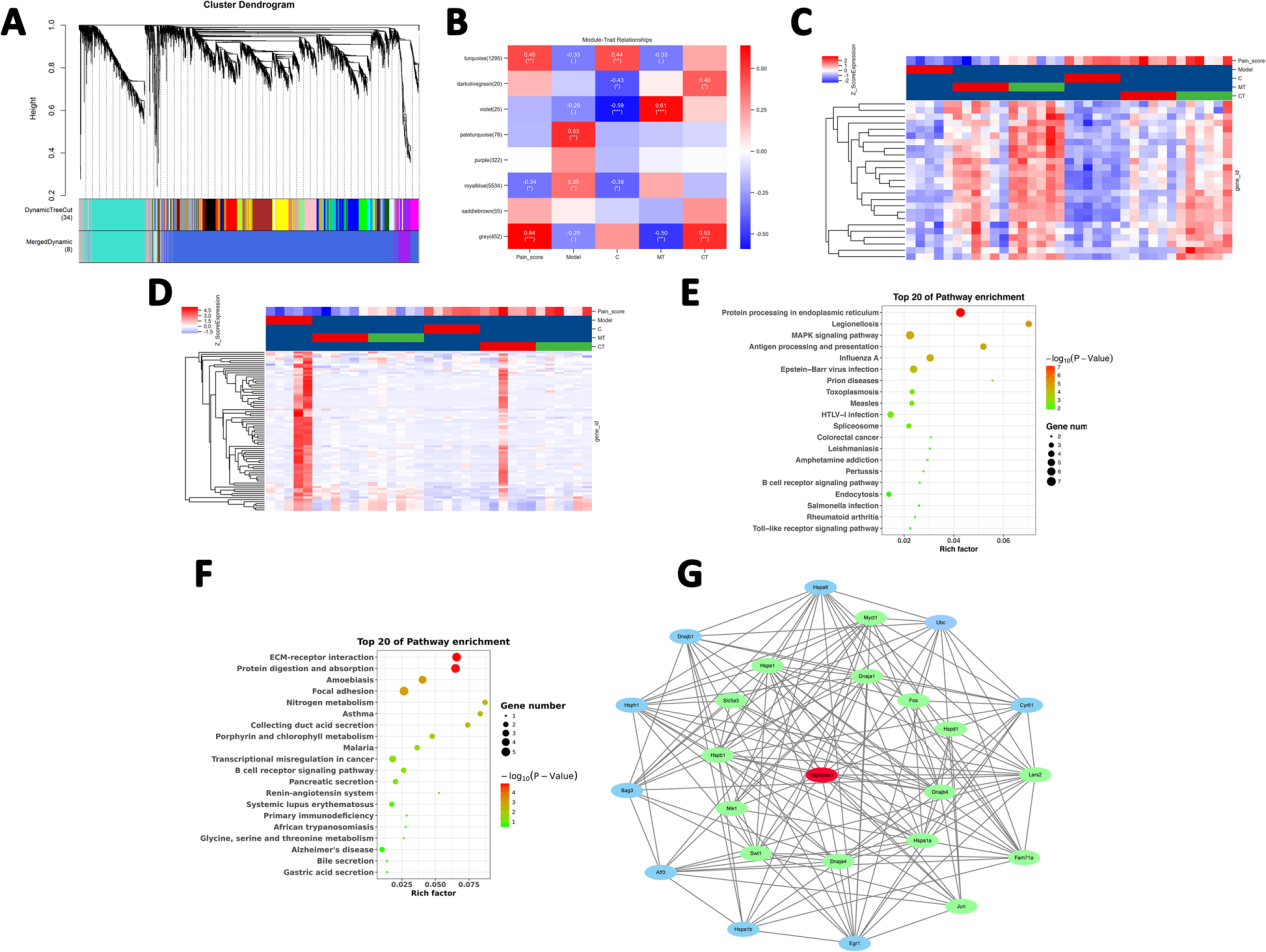
WGCNA analysis. **A** Cluster dendrogram of the co-expression network. The lower part shows the distribution of each module. The same color represents the same module. Each line represents a different gene. The size of modules is proportional to the number of genes. **B** Heat map of modules and correlated traits. The correlation coefficient and *p*-value were shown in each region. Red indicates positive a correlation while blue indicates a negative correlation. The hierarchical clustering of genes in violet (**C**) and paleturquoise module (**D**). KEGG pathway enrichment of genes in violet (**E**) and paleturquoise module (**F**). The circle size represents the number of DEGs, and the color from red to green indicates a gradual decrease in significance. **G** The network of genes in violet module. The Hsp90aa1 played a hub role in the violet module by interacting to multiple proteins of green ellipse

To mine the critical gene co-expression network involved in moxibustion at Zusanli acupoint in AA rat, we performed WGCNA using the raw transcriptomic datasets of all samples. WGCNA is a common technique used to decipher the co-expression pattern among genes [9]. Using WGCNA, we can identify the gene modules and key genes related to moxibustion. In our study, a total of 21,878 raw genes in 35 samples were filtered to remove all genes with low variation in FPKM (SD ≤ 0.3), even for a single replicate of any sampling point of the samples. Eventually, we ended up with 7781 genes in 35 samples and clustered into eight modules (Fig. 6A). Moreover, we analyzed the correlation between network modules and sample characteristics using the Pearson Correlation Coefficient. The violet module was positively correlated with model temperature (MT) while negatively correlated with control (C), and the paleturquoise module was positively correlated with model (M) (Fig. 6B). The expression pattern of violet and paleturquoise module was significant different and shown in Fig. 6C and D. Moreover, the violet module gene was significantly enriched in B cell signaling pathway, MAPK signaling pathway and Rheumatoid arthritis (Fig. 6E). The paleturquoise module gene was involved in B cell signaling and primary immunodeficiency (Fig. 6F). Importantly, we found that Hsp90aa1 was also observed in violet module and played a central role in the violet module by interacting with multiple proteins of green ellipse (Fig. 6G). These results suggested that the critical module violet and paleturquoise were significantly correlated with moxibustion at Zusanli acupoint in AA rat, and the Hsp90aa1 may have played a key role in this process.

## Discussion

Moxibustion is one of the traditional oriental acupuncture therapeutics that heat acupoints by burning dried leaves (such as Artemisia argyi) to treat diseases. Moxibustion can be used to treat many diseases, such as atherosclerotic lesions [10, 11] and RA [4]. In this study, we constructed AA rat induced by CFA, and moxibustion with temperatures 37 °C and 42 °C showed an improving effect on arthritis. Then, we used transcriptome sequencing to characterize the molecular profile of moxibustion warm at 37 °C and 42 °C in the treatment of AA, and combined with WGCNA to uncover key co-expression networks and critical gene of Hsp90aa1 in this process.

In this study, moxibustion with different temperatures (37 °C and 42 °C) generated different analgesic effect, and moxibustion warm temperatures 42 °C was stronger than 37 °C in rat with AA. Previous studies have supported our conclusion that in chronic inflammatory pain induced by CFA, the higher the moxibustion temperature, the better the analgesic intensity, showing 52 °C > 47 °C > 42 °C > 37 °C [12]. However, at present, the molecular basis of moxibustion therapy is still weak. In our study, we found that treated with moxibustion in AA rat, abnormally expressed genes were mainly related to immune and inflammatory pathways, which meant that moxibustion activated the immune system of AA rats. Study proved that moxibustion could inhibit NF-κB by activating Nrf2 and suppress the serum level of IL-1β, IL-6, and IL-8 to alleviate neuroinflammation [13]. Kawanami et al. proved that moxibustion could promote wound healing [14], which is also potentially associated with immune system activation. In addition, mild moxibustion could control NLRP3 inflammasome signaling to alleviate post-inflammatory irritable bowel syndrome [15]. These results supported our conclusion that moxibustion activated immune and inflammatory responses. In the present study, particularly, the MAPK signaling pathway, B cell signaling pathway, and Cytokine-cytokine receptor interaction pathway frequently appeared in the DEG pathway enrichment with different screening methods. MAPK signaling pathway exerted a critical role when eased chronic inflammatory visceral pain with moxibustion in the spinal cord in rats [16]. In addition, although there are few studies on the functions of B cell signaling pathways and Cytokine-cytokine receptor interaction pathways in moxibustion therapy, we know that they are closely related to immunity and inflammation. Therefore, we believe that these DEGs may participate in moxibustion intervention to treat AA through the above pathways.

Fortunately, we identified Hsp90aa1, a key molecule in moxibustion intervention therapy for AA, through transcriptional sequencing, WGCNA, and STC analysis. Hsp90aa1 belongs to the Hsp90 subfamily of the Hsp family and is one of the most important heat shock proteins. As we know, Hsp90 are highly conserved chaperone proteins that play an important role in immune and inflammation [17, 18]. Eckl et al. demonstrated that down-regulation Hsp90 would influence the response of heat-shock and innate immune [19]. Similarly, Hsp90 inhibition suppresses lipopolysaccharide-induced endothelial inflammation [20] and arteriosclerosis inflammation in ApoE mice [21]. In a word, accumulating studies supported that Hsp90 mediates the regulation of immune inflammation in the treatment of multiple inflammatory diseases. However, there is almost no research on Hspaa1 in moxibustion therapy. In the Hsp family, it is clear that moxibustion reduces visceral hyperalgesia by inhibiting the expression of Hsp70 and transient receptor potential vanilloid 1 in rat bone marrow cells [22]. Also, Kobayashi identified that Hsp 70, Hsp 85, and Hhsp 100 were expressed in rats after the stimulation by moxibustion at the hip muscle [23]. Therefore, we speculated that Hsp90aa1 may exert the function of moxibustion therapy as the Hsp family by regulating inflammatory, whereas a large number of clinical studies will provide more evidence for this.

## Conclusions

In conclusion, we demonstrated that moxibustion warm at 37 °C and 42 °C improved CFA - induced AA inflammation, foot swelling, and TWL. This study also profiled the transcriptome-wide landscape to obtain numerous DEGs, and identified 11 significant STC patterns using 3641 DEGs, and constructed the core gene co-expression network using WGCNA, after moxibustion warm at Zusanli acupoint in AA rat, and provided a candidate gene of Hsp90aa1. This study provides a large number of genetic resources and potential molecular evidence for moxibustion warm at Zusanli acupoint treatment of AA.

## Supplementary Information

**Additional file 1 Supplementary Table 1.** Quality preprocessing of transcriptome sequencing data.

## Data Availability

The datasets used and/or analyzed during the current study are available from the corresponding author on reasonable request. The RNA-seq data generated in this study is available in National Center for Biotechnology Information under accession number PRJNA681415.
