# Supplementary material for: Transcriptome sequencing reveals core regulation modules and gene signatures of Zusanli acupoints in response to different moxibustion warm stimulation in adjuvant arthritis rat
Source: Hereditas. 2022 Feb 22;159:15. doi: 10.1186/s41065-022-00221-4 (PMC8862468; doi:10.1186/s41065-022-00221-4)
Supplement: Supplementary file 1 — Additional file 1 Supplementary Table 1. Quality preprocessing of transcriptome sequencing data. [file 41065_2022_221_MOESM1_ESM.docx]

Supplementary Table 1. Quality preprocessing of transcriptome sequencing data.

| Sample | clean_bases | valid_bases | | clean_reads | valid_reads | | Q30% | | GC% | Total mapped reads% |
| --- | --- | --- | --- | --- | --- | --- | --- | --- | --- | --- |
| CM37_1 | 7.87G | 91.40% | 56.11M | | 97.74 | 94.37 | | 49.49 | | 98.84 |
| CM37_2 | 6.92G | 91.40% | 49.38M | | 97.86 | 94.87 | | 49.40 | | 98.75 |
| CM37_3 | 7.72G | 92.21% | 54.53M | | 97.74 | 94.37 | | 49.83 | | 98.7 |
| CM37_4 | 6.83G | 91.73% | 48.47M | | 97.74 | 94.37 | | 49.91 | | 98.64 |
| CM37_5 | 7.69G | 92.47% | 54.30M | | 97.73 | 94.86 | | 49.93 | | 98.78 |
| CM37_6 | 6.81G | 91.65% | 48.47M | | 97.86 | 94.92 | | 50.08 | | 98.75 |
| CM42_1 | 8.02G | 91.24% | 57.35M | | 97.92 | 94.74 | | 49.71 | | 98.62 |
| CM42_2 | 8.03G | 92.16% | 56.92M | | 97.74 | 94.27 | | 50.84 | | 97.87 |
| CM42_3 | 7.69G | 90.59% | 55.23M | | 97.86 | 94.24 | | 50.38 | | 98.53 |
| CM42_4 | 7.39G | 91.32% | 52.76M | | 97.74 | 94.74 | | 49.98 | | 98.65 |
| CM42_5 | 7.16G | 92.71% | 50.51M | | 97.74 | 94.46 | | 49.08 | | 98.43 |
| CM42_6 | 6.88G | 91.88% | 48.87M | | 97.73 | 94.86 | | 50.12 | | 98.63 |
| Con_1 | 8.05G | 92.66% | 56.76M | | 97.86 | 94.72 | | 49.11 | | 98.72 |
| Con_2 | 7.71G | 91.94% | 54.66M | | 97.92 | 94.58 | | 49.33 | | 98.88 |
| Con_3 | 7.80G | 91.15% | 55.68M | | 97.74 | 94.43 | | 50.22 | | 98.82 |
| Con_4 | 7.87G | 91.34% | 56.26M | | 97.86 | 94.77 | | 49.79 | | 98.75 |
| Con_5 | 8.07G | 91.24% | 57.53M | | 97.74 | 94.12 | | 49.60 | | 98.78 |
| Con_6 | 6.80G | 90.54% | 48.92M | | 97.74 | 94.37 | | 49.67 | | 98.76 |
| MM37_1 | 7.91G | 91.85% | 56.17M | | 97.86 | 94.08 | | 49.62 | | 98.54 |
| MM37_2 | 7.30G | 92.72% | 51.49M | | 97.74 | 94.28 | | 49.37 | | 98.54 |
| MM37_3 | 8.00G | 92.16% | 56.70M | | 97.74 | 94.11 | | 49.71 | | 98.26 |
| MM37_4 | 6.74G | 90.86% | 48.52M | | 97.93 | 94.28 | | 49.49 | | 98.68 |
| MM37_5 | 7.26G | 91.79% | 51.66M | | 97.86 | 94.10 | | 48.00 | | 98.68 |
| MM37_6 | 6.44G | 92.75% | 45.31M | | 97.92 | 94.18 | | 49.28 | | 98.76 |
| MM42_1 | 6.95G | 92.56% | 48.97M | | 97.74 | 94.04 | | 49.72 | | 98.56 |
| MM42_2 | 7.97G | 91.83% | 56.57M | | 97.86 | 93.92 | | 50.04 | | 98.42 |
| MM42_3 | 6.96G | 92.95% | 48.94M | | 97.74 | 94.19 | | 49.06 | | 98.66 |
| MM42_4 | 7.33G | 92.40% | 51.80M | | 97.74 | 94.10 | | 49.91 | | 98.54 |
| MM42_5 | 7.37G | 92.64% | 52.01M | | 97.93 | 94.30 | | 49.94 | | 98.63 |
| MM42_6 | 7.17G | 92.14% | 50.83M | | 97.86 | 94.53 | | 50.19 | | 98.64 |
| Model_2 | 7.19G | 92.43% | 50.81M | | 97.92 | 94.22 | | 49.79 | | 98.66 |
| Model_3 | 7.70G | 91.91% | 54.72M | | 97.74 | 94.08 | | 49.45 | | 98.68 |
| Model_4 | 7.77G | 92.61% | 54.85M | | 97.86 | 94.33 | | 48.72 | | 98.78 |
| Model_5 | 6.11G | 92.19% | 43.25M | | 97.74 | 94.04 | | 50.04 | | 98.66 |
| Model_6 | 7.35G | 92.31% | 52.04M | | 97.74 | 94.24 | | 49.48 | | 98.68 |
